# Elevated Proportions of Circulating CXCR5^+^ Follicular Helper T Cells Reflect the Presence of Airway Obstruction in Asthma

**DOI:** 10.1155/2024/2020514

**Published:** 2024-09-19

**Authors:** Tsukie Kin Tsukuda, Kimiko Tsuji, Akari Nishimori, Takehiko Ito, Yuka Kobayashi, Taro Suzuki, Akihito Yokoyama

**Affiliations:** Department of Respiratory Medicine and Allergology Kochi Medical School Kochi University, Kochi, Japan

## Abstract

**Materials and Methods:**

Using flow cytometry, we identified and quantified Group 2 innate lymphocytes, T helper 2 cells, follicular helper T cells, and T helper 17 cells in peripheral blood samples from 49 individuals with asthma. We then conducted cross-sectional analyses to assess relationships between levels of these immune cells and lung function parameters, including the percentage predicted forced expiratory volume in 1 s (%FEV1). We also examined correlations between the proportions of immune cells and type 2 biomarkers.

**Results:**

Proportions of CXCR5^+^ follicular helper T cells in human peripheral blood, as opposed to Group 2 innate lymphoid cells (ILC2) or T helper 2 cells, were significantly higher in cases with %FEV1 < 80% compared to those with %FEV1 ≥ 80%. Further, these proportions correlated negatively with %FEV1 and positively with blood eosinophil counts.

**Conclusions:**

The proportion of circulating follicular helper T cells, but not T helper 2 cells or Group 2 innate lymphoid cells, may reflect the presence of airway obstruction caused by persistent type 2 inflammation.

## 1. Introduction

Maintaining lung function in patients is one of the primary goals of asthma therapy. Asthma is considered a syndrome encompassing a wide spectrum, broadly categorizable into type 2 (T2)-high and T2-low endotypes [1]. Notably, approximately 70% of severe asthma cases are attributed to type 2 inflammation, underlining its significance in disease pathogenesis [2]. The progressive declines in lung function observed over time in asthma are closely linked to persistent type 2 airway inflammation and associated airway remodeling. Traditionally, Group 2 innate lymphocytes (ILC2) and T helper 2 (Th2) cells have been implicated as major contributors to localized type 2 inflammation. However, recent studies have shed light on the involvement of various immune cells in this process. Among these, follicular helper T cells (Tfh) have gained attention for their essential role in facilitating B-cell production of high-affinity antibodies within the germinal center. Evidence suggests a higher proportion of Tfh in the peripheral blood of asthmatic patients compared to healthy controls [3, 4], indicating their potential relevance to the disease pathogenesis.

On the other hand, non-T2 asthma lacks a consistent definition but is generally characterized by the absence of elevated eosinophils or other T2 marker levels. In such cases, Th17-driven inflammation is believed to play a significant role [5, 6]. Elevated levels of interleukin (IL)-17 have been documented in the sputum and serum of patients with severe asthma [7, 8], highlighting the involvement of Th17 cells in disease progression.

However, reports on whether these immune cells are actually involved in remodeling and airway obstruction in human specimens are limited and often at the level of animal studies. In mice, type 2 cytokines, such as IL-4, IL-5, IL-9, and IL-13, are reportedly involved in airway remodeling by promoting infiltration and activation of various cells involved in airway inflammation [9, 10]. ILC2 has been reported to play an important role in wound repair and tissue remodeling by promoting the proliferation of epithelial cells and fibroblasts through high expression of amphiregulin, a member of the epidermal growth factor family in the mouse experiments [11, 12]. The existence of an association between circulating ILC2 levels and airway obstruction in asthmatic patients remains controversial, although several reports have suggested such a link [13, 14]. The purpose of this study was to explore whether the proportions of immune cells in peripheral blood indicate the presence of type 2 inflammation and airway obstruction in asthmatics.

A relationship between eosinophils, among other inflammatory cells infiltrating the airways, and airway remodeling has been reported in many studies in both humans and mice. Eosinophils recruited to the airways exacerbate inflammation by producing and releasing various inflammatory mediators and causing tissue destruction through the action of various cationic eosinophil-derived granule proteins in mice. Eosinophils also produce the profibrotic cytokine transforming growth factor beta (TGF-*β*), which induces peribronchial fibrosis and is thought to be involved in smooth muscle cell hyperplasia [15, 16, 17, 18]. Many reports have examined the relationship between human blood and sputum eosinophil counts and declines in lung function [19, 20, 21, 22]. Longitudinal studies have also reported that elevated blood eosinophil counts are not only involved in accelerated declines in lung function among asthmatic patients [21] but also present a risk factor for the development of obstructive damage in patients without asthma [22].

This cross-sectional study examined the relationship between immune cells, lung function, and type 2 biomarkers to determine whether these immune cells in the peripheral blood of asthmatic patients could provide biomarkers indicative of local type 2 inflammation and airway obstruction. This research endeavored to enhance our understanding of the immune mechanisms underlying asthma pathogenesis and identify potential biomarkers for disease stratification and management.

## 2. Materials and Methods

### 2.1. Study Population and Sample Collection

Forty-nine patients with asthma who visited our hospital between April 2021 and January 2024 were enrolled in this study. Asthma severity was classified according to the Global Initiative for Asthma treatment steps, with adherence to proper inhaler technique [23]. Severe asthma was defined based on European Respiratory Society and American Thoracic Society guidelines [24], with airflow limitation defined as a percentage predicted forced expiratory volume in 1 s (%FEV1) <80%. Patients who were well-controlled at Step 1 or 2 were defined as having mild disease; patients who were well-controlled in Step 3 or 4 and who did not meet the aforementioned definition of severe asthma were classified as having moderate disease. Two patients who were untreated at the time of specimen collection were subsequently considered to have mild disease because they were well-controlled in Step 2. Pulmonary function tests were performed within 1 month before and after specimen collection, and all analysis was under postbronchodilation. For T2 inflammation status, blood eosinophil counts ≥300 cells/*μ*L and/or fraction of exhaled nitric oxide (FeNO) ≥37 ppb were defined as the T2-high population, and others as the T2-low population [25, 26, 27].

To assess the reproducibility of our experiment, immune cell changes were analyzed in five asthmatic patients and one chronic obstructive pulmonary disease (COPD) patient on two occasions (at first visit and 2 years later). Patients remained on the same controller treatment and did not require additional therapy during specimen collection. The severity of COPD was defined based on the GOLD definition [28]. Patient background characteristics are shown in *Supplementary table 2*. Asthma attacks were defined as a condition requiring the administration of systemic corticosteroids for ≥3 days in the 2 years prior to the second specimen collection.

This study was approved by the ethics committee of the Kochi Medical School, Kochi University (approval no. 2020-137). Written, informed consent was obtained from all participants prior to enrollment. This study complied with the Declaration of Helsinki.

### 2.2. Flow Cytometry

About 9 mL of peripheral blood was collected and processed into peripheral blood mononuclear cells (PBMCs) using Lymphoprep™ (Nyegaard & Co., Oslo, Norway) and used for the following experiments. To fix and hemolyze whole blood cells, FACS Lysing Solution (FACS lyse, Becton Dickinson Immunocytometry Systems, San Jose, CA) was added to sample tubes for 15 min at room temperature. MACS buffer (phosphate-buffered saline, pH 7.2; 2 mM ethylenediaminetetraacetic acid; 0.5% bovine serum albumin) was used to suspend the pellet. Whole blood and PBMCs were then placed in tubes and stained with combinations of the following antihuman antibodies and left for 20 min at 4°C in the dark: CD45RA-FITC, clone HI100; CD185-PerCP-Cy5.5, clone J252D4; CD196-PE-Cy7, clone G034E3; CD25-AF700, clone BC96; CD3-APC-Cy7, clone UCHT1; CD4-BV510, clone OKT4; CD127-BV605, clone A819D5; CD5-FITC, clone L17F12; TCR*αβ*-FITC, clone IP26; TCR*γδ*-FITC, clone B1; CD19-FITC, clone HIB19; CD66a,c,e-AF488(FITC), clone ASL-32; CD45-PerCP-Cy5.5, clone HI30; CD294(CRTh2)-PE, clone BM16; CD7-PE-Cy7, clone CD7-6B7; CD56-AF700, clone HCD56; CD161-APC, clone HP-3G10; CD117-BV421, clone 104D2 (BioLegend, San Diego, CA); and CD183-APC, clone IC6/CXCR3 (BD Pharmingen, San Jose, CA). FACS LSRFortessa (BD Biosciences, San Jose, CA) and FlowJo software (Tree Star, Ashland, OR) were used for flow cytometric analyses. Each immune cell was defined based on the expression of surface markers, as follows: ILC2, CD45^+^Lineage^−^CD7^+^CD127^+^CRTh2^+^CD161^+^; Th2, CD3^+^CD4^+^CD45RA^−^CXCR5^−^CD25^low-intermediate^CXCR3^−^CCR6^−^; Tfh, CD3^+^CD4^+^CD45RA^−^CXCR5^+^; and Th17, CD3^+^CD4^+^CD45RA^−^CXCR5^−^CD25^low-intermediate^CXCR3^−^CCR6^+^. We collected up to 10^6^ cells for each sample.

### 2.3. Statistical Analysis

Two-group comparisons were performed using Fisher's exact test, the Mann–Whitney *U*-test, and Student's *t*-test. Correlations were performed using Spearman's rank correlation coefficient and Pearson's product–moment correlation coefficient. Experiments to confirm reproducibility were evaluated using intraclass correlation coefficients (ICC). Statistical significance was set at the level of *P* < 0.05. All statistical analyses were performed using IBM SPSS Statistics version 28 Standard (IBM Corp., Armonk, NY).

## 3. Results

### 3.1. Comparison of Each Item between Patients with and without Airway Obstruction *Clinical Data*

Patients enrolled in this study were divided into two groups: %FEV1 ≥80% and %FEV1 < 80%. No significant differences between groups were seen in terms of age, sex, body mass index (BMI), smoking history, severity, or duration of asthma (Table 1).

### 3.2. Laboratory Data

Regarding the various biomarkers involved in type 2 inflammation, significant differences were seen in blood eosinophil counts (*P* < 0.001), but not FeNO levels or serum IgE levels in asthmatic patients with airway obstruction compared to those without. The frequency of T2-high was 63.3% among the total population but was significantly higher in the group with %FEV1 < 80% (83.3%).

### 3.3. Immune Cells Analyzed by Flow Cytometry

As for immune cells, no significant differences were evident in proportions of ILC2 or Th2 cells between the two groups, but the proportion of Tfh was significantly higher in the group with %FEV1 < 80% (*P*=0.001). In addition, the proportion of Th17 cells was significantly lower in the group with %FEV1 < 80% (*P*=0.037).

### 3.4. Relationships among Immune Cells, Biomarkers, and Airway Obstruction

**Figure 1:**
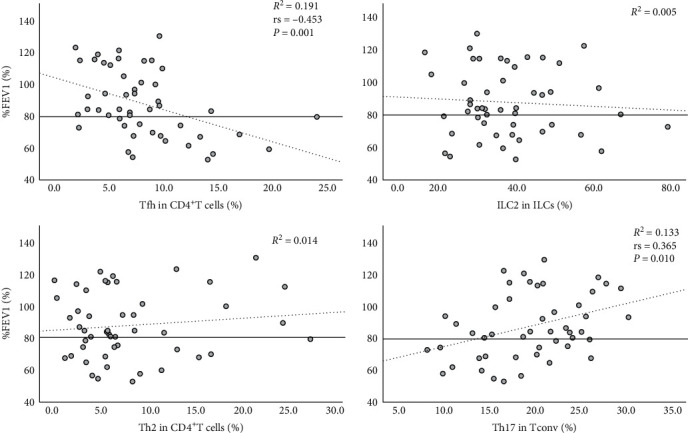
Correlation between the proportion of each immune cell and %FEV1. Clockwise from top left: proportions of Tfh cells, ILC2, Th17 cells, and Th2 cells. *n* = 49. ILC2, Group 2 innate lymphoid cells; Tfh, follicular helper T cells; %FEV1, percentage predictive value of forced expiratory volume in 1 s.

**Figure 2:**
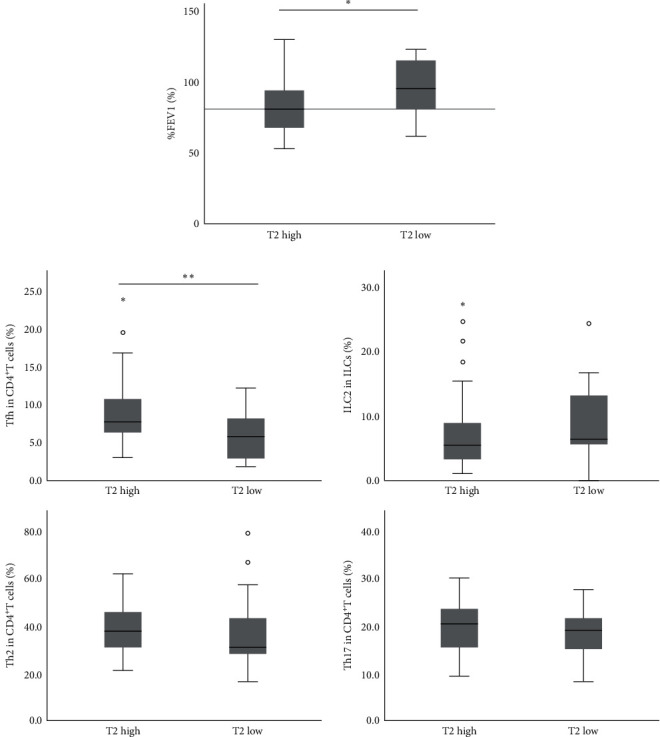
Comparison of %FEV1 and then the proportion of each immune cell with type 2 inflammation status. (a) Comparison of %FEV1 in T2-high and T2-low patients. (b) Comparison of the proportion of each immune cell in T2-high and T2-low patients. The vertical axis shows the proportions of Tfh cells, ILC2, Th2 cells, and Th17 cells from left to right. The numbers of T2-high and T2-low patients are 31 and 18, respectively. %FEV1, percentage predicted forced expiratory volume in 1 s; T2, type 2; ILC2, Group 2 innate lymphoid cells; Tfh, follicular helper T cells.

A negative correlation was identified between %FEV1 and the proportion of Tfh, but no correlations were observed with ILC2 or Th2 cells (Figure 1). In addition, the proportion of Th17 cells showed a weak positive correlation with %FEV1.  Table 2 shows correlations between type 2 biomarkers and the proportions of each immune cell type. Blood eosinophil count showed a positive correlation with the proportion of Tfh, but no significant correlations were observed with ILC2 or Th2 cells. No significant correlation was found between any immune cell types and FeNO levels. No significant correlation was found between serum IgE levels and proportions of Tfh, ILC2, or Th17 cells, but a weak positive correlation was found with the proportion of Th2 cells. The T2-high group showed significantly lower %FEV1 than the T2-low group (Figure 2(a)). The proportion of Tfh was significantly higher in theT2-high group, while other immune cells did not show significant differences between groups (Figure 2(b)).

Other laboratory findings (C-reactive protein, white blood cell count, neutrophils, and lymphocytes) did not show any correlations with the proportions of each immune cell type (Tfh, ILC2, Th2 cell, and Th17 cell). Moreover, the proportion of each immune cell type was compared according to the severity of asthma (mild, moderate, and severe), but no significant differences were observed.

### 3.5. Patient Treatment Details

In terms of controllers used by the 49 patients with asthma in this study, 47 patients utilized inhaled corticosteroids (ICS), with two patients using low doses, 20 using medium doses, and 25 using high doses. In addition, 46, 36, and 25 patients were using long-acting *β*-agonists (LABA), long-acting muscarinic antagonists (LAMA), and leukotriene receptor antagonists (LTRA), respectively. Moreover, six patients had been prescribed oral corticosteroids (OCS), with doses of ≤2.5 mg/day in four patients and 5 and 10 mg/day in one patient each.

### 3.6. Reproducibility of Experiments

In the experiments to validate reproducibility, ICC (1, 1) and 95% confidence intervals for Tfh, ILC2, Th2 cells, and Th17 cells were 0.972 (0.841‒0.996), 0.512 (−0.318‒0.912), 0.963 (0.799‒0.993), and 0.783 (0.157‒0.966), respectively (*Supplementary figure 1*). Results for the proportions of Tfh and Th2 cells were reproducible, while those for ILC2 were less reproducible.

## 4. Discussion

These findings corroborate previous reports, highlighting an association between blood eosinophil count and airway obstruction in asthma. The T2-high population showed a significantly lower %FEV1 than the T2-low population. Notably, in the blood, the proportion of Tfh, but not ILC2 or Th2 cells (widely recognized as major contributors to type 2 inflammation), was associated with the presence of airway obstruction in this study. In addition, the proportions of ILC2 and Th2 cells showed no significant difference between T2-high and T2-low populations, while the proportion of Tfh showed a significant difference between the two. Furthermore, the proportion of Tfh also correlated with blood eosinophil counts.

Tfh play a crucial role in regulating humoral immunity and are vital for proper immune function. In the germinal centers of lymph follicles, Tfh stimulate B cells to produce high-affinity antibodies. Furthermore, circulating CXCR5+ follicular helper T cells are memory Tfh that have migrated from the lymph follicle to defend the host from pathogen reinvasion after priming and may represent precursors of pathogenic effector Th2 cells in the local environment. IL-4-committed Tfh, initially differentiated from naïve T cells stimulated by dendritic cells (DCs) activated by thymic stromal lymphopoietin (*TSLP*), have been reported to differentiate into pathogenic Th2 cells and recruit eosinophils to the lungs after house dust mite sensitization in murine models [29]. In an experiment using primary DCs and naïve CD4^+^ T cells from human blood, the polarity of cells expressing Tfh markers such as CXCR5, programed cell death protein 1 (PD-1), and inducible T-cell costimulator (ICOS) was reportedly induced, with this differentiation dependent on the OX-40 ligand, and about 30% of CD4^+^ T cells activated with TSLP-DC were IL-21-positive and showed strong IL-21 polarity [30]. In addition, Tfh undergoes cell fate determination by expression of the master transcription factor Bcl6 and acquisition of the cell surface-expressed chemokine receptor CXCR5 in the interfollicular zone, leading to early Tfh and promotion of migration from the T-cell zone to the B-cell follicle [31, 32]. Lüthje et al. [33] reported that primed Tfh retain plasticity and can differentiate into effector T cells that migrate to nonlymphoid tissues by secondary challenge. Another report found that Tfh, after the contraction phase of the immune response, becomes memory Tfh, some of which is present in the blood and not only promotes the recall response but also gives rise to other functional effector T cells by plasticity [34].

If Tfh are indeed the precursors of effector Th2 cells that recruit eosinophils to the airways, the identification of conventional Th2 cells among the CXCR5-negative population in this study may be one reason why no association was found between Th2 cells and T2 inflammatory status or airway obstruction. On the other hand, we have previously shown that Th2 cells in bronchoalveolar lavage fluid, identified by the same surface markers in this study, are involved in the progression of pulmonary fibrosis [35]. In that previous study, the proportion of Th2 cells in the local environment was associated with the progression of pulmonary fibrosis, but the proportion of Th2 cells in the blood was not. One possibility is that also in asthma, the proportion of Th2 cells involved in the pathogenesis is increased in the local area but not in the blood.

Next, one possible explanation for why circulating ILC2 levels were not associated with T2 inflammatory status or airway obstruction is that ILC2 are highly plastic and heterogeneous cells [36]. Phenotype and function reportedly vary with the specific tissue in which they reside and even within that tissue [37, 38]. Indeed, even though one pilot study reported that ILC2 from asthmatic patients identified as CD45 + Lineage−CRTH2 + CD127 + CD161 + produced large amounts of IL-5 and IL-13 compared to ILC2 from healthy individuals [39], another study reported that a certain number of human ILCs not expressing CRTH2 (CD 45 + Lineage−) expressed GATA3 and IL-5 by RNA-seq analysis [40].

Another possible cause is that the proportion of circulating ILC2 fluctuates widely from period to period, as evident in the experiment examining reproducibility. Since ILC2 are originally extremely scarce in peripheral blood, even minute changes in the number of ILC2 may be reflected as large changes in proportions. Thus, at present, clearly identifying ILC2 in peripheral blood that may be involved in local type 2 inflammation based on cell surface markers alone may be difficult.

In the present study, patients with airway obstruction had a lower proportion of Th17, which correlated positively with %FEV1. Th17 cells reportedly reach the lungs and increase not only neutrophils but also eosinophils when sensitized and stimulated by allergens, while another study reported that IL-17 suppresses eosinophil differentiation in bone marrow, decreases the number of eosinophils in peripheral blood and bronchoalveolar lavage fluid, and inhibits eosinophilic airway inflammation in mouse experiments [41, 42, 43]. IL-17 is essential for the establishment of eosinophilic airway inflammation during antigen sensitization but may attenuate the response after sensitization. In any case, the present results regarding the association between Th17 and %FEV1 may have been influenced by the fact that patients in this study were predominantly in the T2-high group; the association between Th17 and airway obstruction should be clarified in the T2-low population, but the T2-low population is particularly heterogeneous and is expected to be more difficult to analyze than the T2-high population.

Limitations of this study include the low number of patients from a single institution, the lack of functional analysis of Tfh and other immune cells, and that %FEV1 data, which vary over time, were obtained only once in this cross-sectional investigation. However, no reports to date have determined the relationship between circulating Tfh in human blood samples and lung function.

## 5. Conclusion

In conclusion, the proportion of circulating Tfh, as opposed to ILC2 or Th2 cells, may reflect the presence of airway obstruction in asthmatic patients caused by persistent type 2 inflammation.

## Figures and Tables

**Table 1:** Patient characteristics and comparison of each item with percentage predicted value of FEV1 greater than 80% and less than 80%.

Percentage of predicted value of FEV1	All patients, *n* = 49	≥80%, *n* = 31	<80%, *n* = 18	*P* value
Clinical information				
Age (years)	62.6 ± 11.7	61.5 ± 12.1	64.4 ± 10.8	0.202
Sex, male (*n* (%))	15 (30.6)	10 (32.3)	5 (27.8)	1.000
BMI (kg/m^2^)	24.5 ± 4.8	25 ± 5.2	23.6 ± 4.0	0.372
Smoking status (*n*) never/former/current	32/12/5	20/8/3	12/4/2	1.000
Severity (mild/moderate/severe)	3/21/25	3/16/12	0/5/13	0.056
Asthma duration of disease (months)	214.8 ± 156.4	207.1 ± 156.5	228 ± 155.2	0.660
Spirometry				
%FEV1 (%)	87.6 ± 20.6	99.9 ± 15.0	66.3 ± 7.8	<0.001^†^
Laboratory test findings				
CRP (mg/dL)	0.199 ± 0.236	0.161 ± 0.193	0.245 ± 0.272	0.340
WBC (×10^3^/*μ*L)	6.97 ± 2.74	7.13 ± 3.10	6.72 ± 3.10	0.878
Neutrophils (×10^3^/*μ*L)	4.29 ± 2.42	4.49 ± 2.78	3.98 ± 1.71	0.635
Lymphocytes (×10^3^/*μ*L)	1.89 ± 6.10	1.94 ± 0.617	1.81 ± 0.59	0.610
Serum biomarkers of type 2 inflammation				
Serum IgE (IU/mL)	851.9 ± 1,553.4	684 ± 1,039.6	1,128.3 ± 2,117.6	0.558
Blood eosinophil count (/*μ*L)	375.5 ± 283.0	263.3 ± 180.8	568.8 ± 320.4	<0.001^†^
FeNO (bbp)	47.7 ± 49	42.3 ± 42.5	57 ± 57.4	0.513
T2 inflammation status (number in the high group (%))	31 (63.3)	16 (51.6)	15 (83.3)	0.034^*∗*^
Proportion of each immune cell				
Tfh in CD4^+^T cells (%, median (IQR))	7.31 (5.24)	6.83 (5.09)	9.89 (6.67)	0.001^§^
Th2 in Tconv (%, median (IQR))	36.1 (14.6)	36 (14.2)	37.8 (14.93)	0.748
ILC2 in ILCs (%, median (IQR))	5.74 (6)	5.74 (5.47)	6.01 (7.28)	0.868
Th17 in Tconv (%, median (IQR))	20.1 (8.1)	20.8 (7.25)	17.15 (7.35)	0.037^*∗*^

^*∗*^*P* < 0.05 compared with %FEV1 ≥ 80% group, ^§^*P* < 0.01 compared with %FEV1 ≥ 80% group, ^†^*P* < 0.001 compared with %FEV1 ≥ 80% group. Unless otherwise stated, data are presented as mean ± SD. BMI, body mass index; %FEV1, percentage predicted forced expiratory volume in 1 s; CRP, C-reactive protein; WBC, white blood cell; IQR, interquartile range; ILCs, innate lymphoid cells; ILC2, Group 2 innate lymphoid cells; Tfh, follicular helper T cells; ICS, inhaled corticosteroids; Tconv, conventional T cells; FeNO, fraction of exhaled nitric oxide; %FEV1, percentage predicted forced expiratory volume in one second; IgE, immunoglobulin E.

**Table 2:** Correlation between type 2 biomarkers and each immune cell.

Proportion of:	FeNO level (ppb)		Blood eosinophil count (/*μ*L)		Serum IgE level (IU/mL)	
	Correlation coefficient (rs)	*P* value	Correlation coefficient (rs)	*P* value	Correlation coefficient (rs)	*P* value
Tfh in CD4^+^T cells (%)	0.120	0.411	0.498	<0.001	−0.140	0.360
ILC2 in ILCs (%)	−0.117	0.421	−0.109	0.455	0.167	0.272
Th2 in Tconv (%)	−0.180	0.216	0.035	0.814	0.334	0.025
Th17 in Tconv (%)	0.164	0.260	−0.251	0.082	0.089	0.563

ILCs, innate lymphoid cells; ILC2, Group 2 innate lymphoid cell; Tfh, follicular helper T cells; Tconv, conventional T cells; FeNO, fraction of exhaled nitric oxide.

## Data Availability

Data and protocols are available upon request from the corresponding author.

## Abbreviations

T2: type 2
ILC: innate lymphoid cells
ILC2: group 2 innate lymphoid cells
Th2: T helper 2
Th17: T helper 17
TGF-*β*: transforming growth factor beta
ICS: inhaled corticosteroid
LABA: long-acting *β*-agonist
LAMA: long-acting muscarinic antagonist
LTRA: leukotriene receptor antagonist
OCS: oral corticosteroid
PSL: prednisolone
BMI: body mass index
COPD: chronic obstructive pulmonary disease
PBMCs: peripheral blood mononuclear cells
Tfh: follicular helper T cells
Tconv: conventional T cells
ICC: intraclass correlation coefficients
CRP: C-reactive protein
WBC: white blood cell
FeNO: fraction of exhaled nitric oxide
IgE: immunoglobulin E
FEV1: forced expiratory volume in 1 s
%FEV1: percentage predicted forced expiratory volume in 1 s
DCs: dendritic cells
TSLP: thymic stromal lymphopoietin
PD-1: programed cell death protein 1
ICOS: inducible T-cell costimulatory.

## Additional Points

*Key Message*. The proportion of circulating Tfh was elevated in asthmatic patients with airway obstruction more than in those without. Proportions of circulating ILC2 and Th2 did not reflect T2 inflammation or airway obstruction. The proportion of circulating Tfh correlated positively with blood eosinophil counts.

## Supplementary Materials

Supplementary 1 Figure 1: experimental results to verify reproducibility.

Supplementary 2 Table 1: background characteristics of the six patients for experiments to confirm reproducibility.
