# Supplementary figures and images for: Elevated Proportions of Circulating CXCR5+ Follicular Helper T Cells Reflect the Presence of Airway Obstruction in Asthma
Source: J Immunol Res. 2024 Sep 19;2024:2020514. doi: 10.1155/2024/2020514 (PMC11427719; doi:10.1155/2024/2020514)

**Figure S1.** Experimental results to verify reproducibility

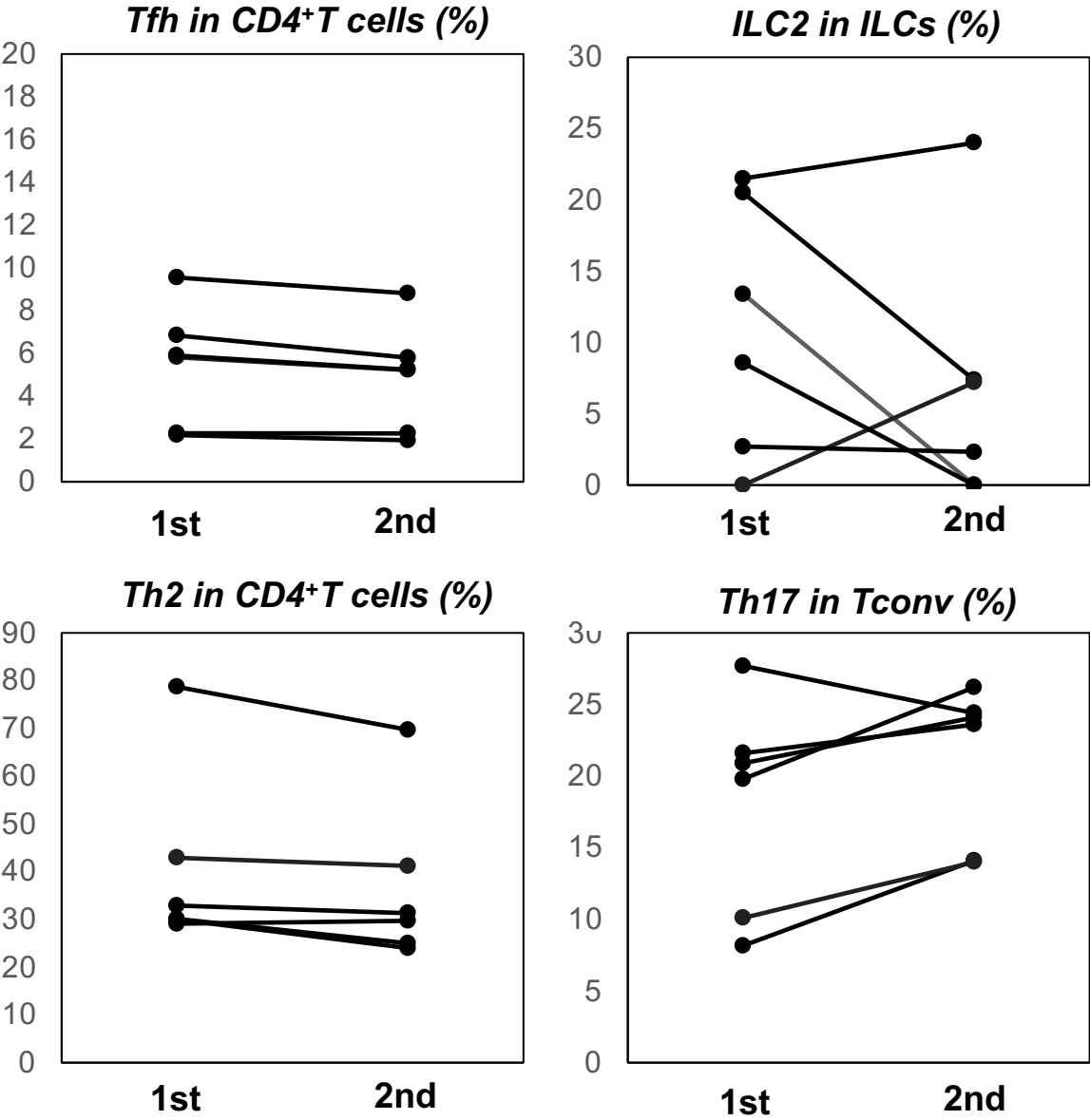

Supplement: Supplementary 1 — Figure 1: experimental results to verify reproducibility. [file 2020514.f1.pdf]
