# Supplementary material for: Elevated Proportions of Circulating CXCR5+ Follicular Helper T Cells Reflect the Presence of Airway Obstruction in Asthma
Source: J Immunol Res. 2024 Sep 19;2024:2020514. doi: 10.1155/2024/2020514 (PMC11427719; doi:10.1155/2024/2020514)
Supplement: Supplementary 2 — Table 1: background characteristics of the six patients for experiments to confirm reproducibility. [file 2020514.f2.docx]

**Table S1. Background characteristics of the six patients for experiments to confirm reproducibility**

ICS, inhaled corticosteroids; LABA, long-acting β-agonist; LAMA, long-acting muscarinic antagonist; LTRA, leukotriene receptor antagonist; FeNO, fraction of exhaled nitric oxide; Eos, eosinophil count; M, male; F, female. COPD, chronic obstructive pulmonary disease; F, female; %FEV1, percent predicted forced expiratory volume in one second.

| **Treatment** | | High-dose ICS, LABA, LAMA and LTRA | Medium-dose ICS, LABA, LAMA, and LTRA | Medium-dose ICS and LABA | Medium-dose ICS and LABA | Medium-dose ICS, LABA, and LAMA | Medium-dose ICS, LABA, and LAMA |
| --- | --- | --- | --- | --- | --- | --- | --- |
| **%FEV1 (%)** | **2nd** | 135.8 | 87.7 | 127 | 114.3 | 82.8 | 87.5 |
|  | **1st** | 114.7 | 72.8 | 129.8 | 115.7 | 86.5 | 84.2 |
| **FeNO (ppb)** | **2nd** | 9 | 18 | 28 | 5 | 11 | 33 |
|  | **1st** | 11 | 8 | 39 | 10 | 15 | 29 |
| **Eos count (/μL)** | **2nd** | 180 | 100 | 520 | 0 | 70 | 180 |
|  | **1st** | 160 | 170 | 500 | 10 | 0 | 150 |
| **Disease and Severity** | | Severe asthma | Severe asthma | Moderate asthma | Moderate asthma | Mild COPD (GOLD1) | Moderate asthma |
| **Age (y)/Sex** | | 62/F | 79/F | 32/F | 45/F | 58/F | 72/F |
| **Case** | | 1 | 2 | 3 | 4 | 5 | 6 |
